# Gender difference in carotid intima-media thickness in type 2 diabetic patients: a 4-year follow-up study

**DOI:** 10.1186/1475-2840-11-51

**Published:** 2012-07-16

**Authors:** Bo Zhao, Yanping Liu, Yifei Zhang, Yuhong Chen, Zhifang Yang, Ying Zhu, Weiwei Zhan

**Affiliations:** 1 Department of Ultrasonography, Ruijin Hospital, Shanghai Jiaotong University Medical School, 197 Ruijin 2nd Road, Shanghai, 200025, People's Republic of China; 2 Shanghai Clinical Center for Endocrine and Metabolic Diseases and Division of Endocrine and Metabolic Diseases of E-Institutes of Shanghai Universities, Ruijin Hospital, Shanghai Jiao Tong University School of Medicine, 197 RuiJin 2nd Road, Shanghai, 200025, People's Republic of China

**Keywords:** Type 2 diabetes mellitus, Intima-media thickness, Gender difference

## Abstract

**Background:**

Different population studies have reported gender difference in carotid intima-media thickness (CIMT), which is proved to be a risk factor of cardiovascular diseases. However, few longitudinal researches examine this gender difference in type 2 diabetes mellitus (T2DM) patients. Therefore, we prospectively analyzed CIMT in T2DM patients over a 4-year follow-up period.

**Methods:**

355 T2DM patients (mean age 59 years; 54.9% women) were included in the present study. CIMT were measured using Color Doppler ultrasound. CIMT was measured at baseline (CIMT) in 2006 and at follow-up in 2010. Biochemical and clinical measurements were collected at baseline.

**Results:**

Mean value of CIMT1 and CIMT2 were 0.740 ± 0.148 mm and 0.842 ± 0.179 mm, respectively. Men had higher CIMT than women both at baseline and at follow-up (CIMT1: 0.762 ± 0.149 vs 0.723 ± 0.146 mm, *P* = 0.0149; CIMT2: 0.880 ± 0.189 vs 0.810 ± 0.164 mm, *P* = 0.0002). Mean annual progression of CIMT (dCIMT) was 0.025 ± 0.022 mm/year. dCIMT was larger in men than in women (0.030 ± 0.025 vs 0.022 ± 0.019 mm, *P* = 0.0006). In multiple regression analyses, age was an independent risk factor of CIMT in both genders, while dCIMT was associated with age only in men.

**Conclusions:**

Gender difference in CIMT was confirmed in T2DM patients. Moreover, impact of ageing on CIMT progression only existed in men, which might be the reason that gender difference in CIMT increased with age.

## Background

Patients with type 2 diabetes mellitus (T2DM) have 2–6 fold higher risk of cardiovascular disease (CVD) than non-diabetics [1], which accounts for nearly 50% diabetes-related mortality [2]. Carotid intima-media thickness (CIMT) measured by ultrasound has been proved to be an independent risk factor of CVD [3,4]. It is suitable for large population study, because this measurement is non-invasive, low-cost and convenient. Moreover, CIMT has been used in clinical trials to evaluate the efficacy of anti-diabetic, anti-hyperlipidemia and anti-hypertensive treatments [5-8].

Cross-sectional population studies have demonstrated that men usually have higher CIMT than women of similar age [9-11]. However, gender difference in CIMT has been seldom studied in longitudinal studies, especially in T2DM patients [12-17].

Therefore, we prospectively analyzed CIMT in T2DM patients over a 4-year follow-up period in aim to investigate gender difference in CIMT and to track CIMT progression.

## Methods

### Subjects

The Ethics Committee of Ruijin Hospital, Shanghai Jiaotong University School of Medicine approved the study protocol. From October 2006 to February 2007, 901 T2DM patients (diagnosed according to the 1999 World Health Organization criteria [18]) were recruited via the outpatient clinic of the Department of Endocrine and Metabolism at Ruijin Hospital, Shanghai. All study participants gave written informed consent. Among them, 501 patients underwent carotid ultrasonographic examination both at baseline (2006) and at follow-up (2010). Among them, 68 patients were excluded because of missing clinical information and/or biochemical indices. Both female and male patients were divided into three groups according to their baseline age: ≤55 years, 56–64 years and ≥65 years (at follow-up: ≤59 years, 60–68 years and ≥69 years). We excluded 78 patients in order to make other potential risk factors of CIMT (including age, body mass index [BMI], systolic and diastolic blood pressure, serum concentrations of high density lipoprotein [HDL] and low density lipoprotein [LDL] cholesterol, total cholesterol, triglycerides, plasma glycosylated hemoglobin A1c [HbA1c], smoking and drinking alcohol) comparable between men and women in each age group. Thus, the present analysis included 355 subjects.

### Clinical and biochemical assessments

Blood pressure was measured using a standard mercury sphygmomanometer in the sitting position after the patients had rested for at least 10 min. Body mass index was calculated as weight in kilograms divided by height in meter squared. A standardized questionnaire was used to collect information on medical history including the duration of type 2 diabetes mellitus and lifestyle.

Blood samples were obtained after an overnight fasting. Plasma HbA1c was measured by high-performance liquid chromatography (BRO-RAD Company, USA). Serum concentrations of total cholesterol and triglycerides were measured by the enzymatic method, and high density lipoprotein (HDL) cholesterol was measured using a specific precipitation method (Beckman LX-20, Brea, CA, USA). Low density lipoprotein (LDL) cholesterol was calculated using the Friedewald’s formula [19].

Cardiovascular disease was defined as angina, myocardial infarction or revascularization. Microvascular disease was defined as diabetic retinopathy, nephropathy or neuropathy.

### CIMT measurement

**Figure 1:**
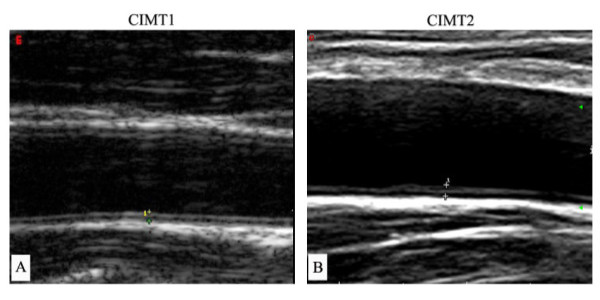
**B-mode tomographic ultrasound figures at CIMT1 and CIMT2.** The distance between two cross marks: carotid intima-media thickness (CIMT). (**A**: using Esaote DU3, Italy in 2006; **B**: using Esaote Mylab90, Italy in 2010).

In 2006 and 2010, the same trained sonographers, who were unaware of the clinical and biochemical information of the subjects, performed CIMT measurements manually using a high-resolution B-mode tomographic ultrasound system (Esaote DU3 and Mylab90, Italy) with a linear 7.5-10 MHz transducer (Figure 1). Precision of the CIMT measurement is 0.01 mm. The sonographers measured CIMT on the fall wall of the right and left common carotid arteries, 1.5 cm proximal to the bifurcation. The transducer was manipulated so that the lumen diameter was maximized in the longitudinal plane. The first and second lines represent the lumen–intimal interface and the collage-contained upper layer of tunic adventitia, respectively [20]. The mean value of the right and left common carotid IMT was used for analysis. The baseline and follow-up CIMT were recorded as CIMT1 and CIMT2, respectively. Progression of CIMT was calculated as the difference between CIMT1 and CIMT2 divided by 4 years and recorded as dCIMT. We performed reproducibility study in 20 subjects. The coefficient of variations were less than 5.1%.

### Statistical analysis

For database management and statistical analysis, we used SPSS version 13.0. Variables with a skewed distribution including systolic and diastolic blood pressure, HbA1c, TG, and HDL cholesterol were normalized by logarithmic transformation. Comparisons of means and proportions were performed with analyses of variance (ANOVA) and Chi-square test, respectively. Univariate correlations were evaluated by Pearson’s and Spearman’s analyses. We searched for possible covariables of CIMT, using a stepwise regression procedure with the P-values for independent variables to enter and to stay in the model set at 0.05. As covariables, we considered age, BMI, systolic and diastolic blood pressure, HDL and LDL cholesterol, total cholesterol, triglycerides, HbA1c, smoking and alcohol intake.

## Results

### Characteristics of the patients

355 subjects were included in the present study (Table 1). Clinical and biochemical variables at baseline were not significantly different between men and women. During a 4-year follow-up, 39 patients (women vs men 26 vs 13, P = 0.13) had cardiovascular events and 115 patients (68 vs 47, P = 0.30) developed microvascular diseases.

**Table 1:** Characteristics of patients at baseline

Characteristics	Men	Women	*P*
Number	160	195	
Age (years)	59.1 ± 9.5	58.5 ± 7.9	0.47
Duration (years)	7.0 ± 5.9	7.8 ± 5.7	0.18
Body mass index (kg/m^2^)	24.4 ± 2.8	24.3 ± 3.4	0.73
Systolic blood pressure (mmHg)	122.2 ± 10.8	124.6 ± 14.6	0.10
Diastolic blood pressure (mmHg)	78.2 ± 7.4	77.2 ± 8.6	0.25
HbA1c (%)	6.7 ± 1.1	6.7 ± 1.2	0.99
Total cholesterol (mmol/L)	5.0 ± 0.9	5.1 ± 0.8	0.09
Triglycerides (mmol/L)	1.4 ± 1.0	1.5 ± 0.8	0.40
HDL cholesterol (mmol/L)	1.5 ± 0.3	1.5 ± 0.2	0.05
LDL cholesterol (mmol/L)	3.0 ± 0.7	3.1 ± 0.6	0.23
Smoking (%)	17.2	18.5	0.76
Alcohol intake (%)	10.2	7.7	0.44
History of hypertension (%)	51.9	53.8	0.71
History of hyperlipidemia (%)	40.6	43.1	0.64
Anti-diabetic treatment (%)	81.9	83.6	0.67
Anti-hyperlipidemia treatment (%)	13.8	20.0	0.12
Anti-hypertensive treatment (%)	51.3	51.3	0.99
Cardiovascular disease (%)	8.1	13.3	0.13
Microvascular disease (%)	29.4	34.9	0.31

Abbreviations: *HDL* high density lipoprotein, *LDL* low density lipoprotein.

### Carotid intima-media thickness

#### CIMT

**Figure 2:**
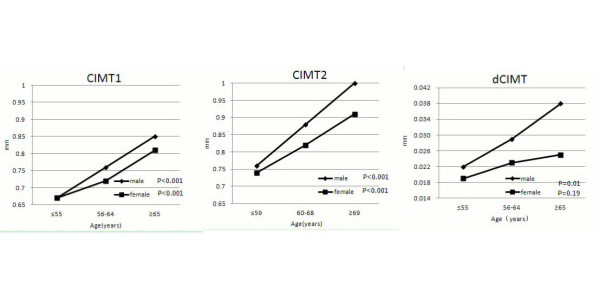
Carotid intima media-thickness at baseline (CIMT1) and follow-up (CIMT2), and carotid intima-media thickness progression (dCIMT) by sex and age groups.

CIMT2 were higher than CIMT1 both in women and in men (Table 2). At baseline and follow-up, men had larger CIMT than women. Figure 2 showed CIMT by gender and age groups. Both female and male patients were divided into three groups according to their baseline age: ≤55 years (follow-up: ≤59 years) (men 46, women 66), 56–64 years (60–68 years) (men 66, women 87) and ≥65 years (≥69 years) (men 48, women 42). In each age group, clinical and biochemical measurements did not have significant differences between women and men. CIMT1 and CIMT2 increased across the age groups in both genders (*P* < 0.001). Men had higher CIMT2 than women in the group with age ≥69 years at follow-up (*P* < 0.05, Figure 2). After adjustment for other covariables, age was associated with CIMT1 in both genders, while BMI was associated with CIMT1 in women (Table 3).

**Table 2:** Carotid intima-media thickness at baseline and follow-up

Characteristics	Men	Women	*P*
CIMT1 (mm)	0.762 ± 0.149	0.723 ± 0.146	0.0149
CIMT2 (mm)	0.880 ± 0.189	0.810 ± 0.164	0.0002
dCIMT (mm/year)	0.030 ± 0.025	0.022 ± 0.019	0.0006

Abbreviations: *CIMT1* Carotid intima-media thickness at baseline, *CIMT2* carotid intima-media thickness at follow-up, *dCIMT* progression in carotid intima-media thickness, it was calculated as (CIMT2-CIMT1)/4 years.

**Table 3:** Correlates of carotid intima-media thickness

	CIMT1		dCIMT	
	Standardized coefficient Beta	*P*	Standardized coefficient Beta	*P*
Women				
Age (years)	0.373	<0.001		
Body mass index (kg/m^2^)	0.192	0.008		
Men				
Age (years)	0.508	<0.001	0.194	0.031

The covariables considered for entry into the multiple regression model were age, body mass index, systolic and diastolic blood pressure, high density and low density lipoprotein cholesterol, total cholesterol, triglycerides, smoking and drinking.

#### dCIMT

Compared with men, women had lower dCIMT (Table 2). In the same age group, dCIMT was higher in men than in women, but significant difference only existed in the group with age ≥65 years (*P* < 0.05, Figure 2). The association between age and dCIMT was observed only in men (*P* = 0.01, Figure 2). After adjustment for other covariables, this association remained significant (*P* = 0.031, Table 3).

## Discussion

The present study found that T2DM men had higher CIMT than T2DM women, especially in those with age ≥ 69 years. Age was the main risk factors of CIMT in both genders. However, the impact of ageing on CIMT progression only existed in male patients.

The gender difference in CIMT in T2DM patients was in line with other population researches. In a study of healthy Taiwan population, CIMT was significantly higher in men than in women (0.558 vs 0.527 mm, *P* = 0.012) [21]. Kablak-Ziembicka et al confirmed this result in subjects without CVD (men *vs* women 1.05 vs 0.93 mm, *P* < 0.001) [22]. CIMT in our study was higher than that in Taiwan healthy population, while was lower than that in population without CVD [22] or with normal glucose tolerance [23]. This might be due to the differences in age, ethnicity and physical condition of study populations.

We found that gender difference in CIMT only existed in the group with age ≥ 69 years. This finding was consistent with the Tromsø study [24], which found that gender difference in CIMT was not significant in young population and gradually increased with age [24]. Dalla Pozza et al reported that there CIMT was not different between male and female children with type 1 diabetes mellitus [25]. However, contrary results were found in subjects without CVD risk factors studied by Sinning et al [26]. In their study, gender difference in CIMT only exists in young population (mean age: 35 years) but not in elderly population (age range: 55–72 years) [26]. The possible reason might be that the estrogen, which suppressed the progression of arteriosclerosis, decreased in postmenopausal women. Thus, gender difference in CIMT was weakened in elderly population as observed by Sinning et al. However, our subjects were patients with diabetics, which is an important and independent risk factor of CVD. Hayashi et al reported gender difference in CVD risk factors [27], which may cause gender difference in CIMT. Moreover, men are more susceptible to CVD risk factor than women [28-30]. Therefore, gender difference in CIMT was significant in our study due to diabetic status.

Another main finding of our study was that CIMT progression was faster in men than women, which was in keeping with other studies [24,31]. Stensland-Bugge et al reported that the average progression per year of CIMT was greater in men than in women (0.010 mm versus 0.009 mm, *P* < 0.01) [24]. In the present study, these value were 0.030 mm for men and 0.022 mm for women (*P* < 0.01). The progression of CIMT in our study was higher than those reported from healthy population (0.007 mm/year in Germen, 0.008 mm/year in Japanese) [32,33] and similar to those reported from subjects with impaired glucose tolerance [34] and T2DM [35]. In the current study, after adjustment for other covariables, age was an independent risk factors of CIMT progression only in men. This might be the reason that men had higher CIMT than women and this gender difference was more significant in patients with old age and more significant after a 4-year follow-up.

The present study must be interpreted within the context of its limitations. First, we did not find gender difference in the incidence of cardiovascular diseases or microvascular diseases after a 4-year follow-up, although men had higher CIMT than women at baseline. This might be due to the small sample size and the relatively short follow-up period. Second, we did not categorize anti-diabetic treatment, which might have effect on the results.

## Conclusion

Our study showed that age was the most important and independent risk factor of CIMT in diabetics. However, impact of ageing on CIMT progression only existed in T2DM men, which might lead to the phenomenon that gender difference in CIMT increased with age.

## Abbreviations

CIMT: Carotid intima-media thickness; dCIMT: Progression of carotid intima-media thickness; T2DM: Type 2 diabetes mellitus; CVD: Cardiovascular disease; SBP: Systolic blood pressure; DBP: Diastolic blood pressure; TG: Triglycerides; TC: Total cholesterol; HDL: High density lipoprotein; LDL: Low density lipoprotein; HbA1C: Glycosylated hemoglobin A1C; BMI: Body mass index.

## Competing interests

The authors declared that they have no conflicts of interests.

## Authors’ contributions

BZ and WZ participated in the design of the study. BZ and YL drafted the manuscript. BZ and YZ analysed the data. BZ, WZ, YZ, YC, ZY, and YZ conceived of the study, and participated in its design and coordination and helped to draft the manuscript. All authors read and approved the final manuscript.
